# Progress and recognition of idiopathic intracranial hypertension: A narrative review

**DOI:** 10.1111/cns.14895

**Published:** 2024-08-04

**Authors:** Chenxia Zhou, Yifan Zhou, Lu Liu, Huimin Jiang, Huimin Wei, Chen Zhou, Xunming Ji

**Affiliations:** ^1^ Department of Neurology Xuanwu Hospital, Capital Medical University Beijing China; ^2^ Neurology and Intracranial Hypertension and Cerebral Venous Disease Center National Health Commission of China, Xuanwu Hospital, Capital Medical University Beijing China; ^3^ Laboratory of Brain Disorders, Ministry of Science and Technology, Collaborative Innovation Center for Brain Disorders Beijing Institute of Brain Disorders, Beijing Advanced Innovation Center for Big Data‐based Precision Medicine, Capital Medical University Beijing China; ^4^ Zhongguancun Xirui Institute of Precision Medicine for Heart and Brain Tumors Beijing China; ^5^ Beijing Advanced Innovation Center for Big Data‐Based Precision Medicine School of Biological Science and Medical Engineering, Beihang University Beijing China; ^6^ Department of Neurosurgery Xuanwu Hospital, Capital Medical University Beijing China

**Keywords:** 11β‐hydroxysteroid dehydrogenase type 1 (11β‐HSD1), glucagon‐like peptide‐1 (GLP‐1), idiopathic intracranial hypertension (IIH), intracranial pressure (ICP), venous sinus stenting (VSS)

## Abstract

**Background:**

Idiopathic intracranial hypertension (IIH) mainly affects obese young women, causing elevated intracranial pressure, headaches, and papilledema, risking vision loss and severe headaches. Despite weight loss as the primary treatment, the underlying mechanisms remain unclear. Recent research explores novel therapeutic targets.

**Aims:**

This review aimed to provide a comprehensive understanding of IIH's pathophysiology and clinical features to inform pathogenesis and improve treatment strategies.

**Methods:**

Recent publications on IIH were searched and summarized using PubMed, Web of Science, and MEDLINE.

**Results:**

The review highlights potential pathomechanisms and therapeutic advances in IIH.

**Conclusion:**

IIH incidence is rising, with growing evidence linking it to metabolic and hormonal disturbances. Early diagnosis and treatment remain challenging.

## Graphical abstract

We integrate recent studies and findings to provide a thorough and detailed exploration of various facets of idiopathic intracranial hypertension, including its epidemiology, pathophysiological mechanisms, clinical features, and treatment strategies. This contributes to a better understanding of the disease and potential therapeutic targets.

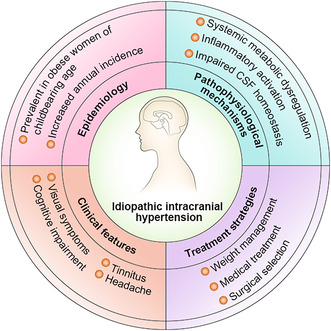

## 1 INTRODUCTION

Idiopathic intracranial hypertension (IIH) is a clinical syndrome characterized by elevated intracranial pressure (ICP) in the absence of vascular or space‐occupying lesions, and without enlargement of the cerebral ventricles, for which no identifiable causative factor can be determined. IIH often manifests as headaches, severe visual impairment, or even blindness.^1^ Recent studies have indicated an increasing trend in the sex‐adjusted and age‐adjusted annual incidence of IIH, currently estimated at 2.4 per 100,000. This condition occurs more frequently in females and individuals with obesity.^2^ Common symptoms present in most patients with IIH include headaches, visual impairment, and pulsatile tinnitus.^3^ However, the pathophysiological mechanisms underlying IIH remain unclear.^4^ Dysregulation of ICP, a key focus of investigation, has been suggested to be caused by disordered cerebrospinal fluid (CSF) dynamics and elevated venous sinus pressure.^5^ Management strategies to relieve intracranial hypertension, primarily through weight loss, may include emergency surgery to preserve vision and pharmacological interventions to minimize headaches.^6^ Recent mechanistic studies have revealed potential new therapeutic targets, including glucagon‐like peptide‐1 receptor (GLP‐1R) and 11β‐hydroxysteroid dehydrogenase type 1 (11β‐HSD1), for IIH treatment.^7^, ^8^ This review summarizes the current understanding of the epidemiology, pathogenesis, clinical manifestations, diagnostic criteria, and management strategies for IIH, as derived from recent studies.

## 2 EPIDEMIOLOGY

Significant heterogeneity has been observed in the epidemiology of IIH in various countries and across different studies, with a pooled annual incidence rate ranging from 0.5 to 3.2 cases per 100,000 individuals (Table 1).^21^, ^22^ IIH is most frequently diagnosed in pregnant women and the population with obesity, with an incidence rate of approximately 7.9–21.4/100,000 per year and an average age of onset of 29 years.^21^ In developed countries, the incidence of IIH increases markedly in parallel with the prevalence of obesity, as evidenced by extensive cohort‐ and population‐based studies. A recent extensive retrospective cohort study that analyzed data from the Welsh Secure Anonymized Information Linkage database between 2003 and 2017 estimated that the prevalence and incidence of IIH are 76/100,000 and 7.8/100,000 per year, respectively.^19^ Similarly, a national case‐control study conducted in Sweden, which compared incidence rates in the first 6 years to those in the last 5 years within the study period, showed a 79% increase in the incidence of IIH.^23^ A comprehensive analysis of a large cohort derived from primary healthcare records in the United Kingdom revealed that the prevalence and incidence of IIH in females were 79/100,000 and 9.3/100,000 per year, respectively. In particular, the highest incidence was observed among females of reproductive age, specifically those aged 20–29 years, at a rate of 16.5 per 100,000 per year.^24^ A meta‐analysis by McCluskey et al. showed that country‐specific IIH incidence, such as in the United Kingdom, correlated with the corresponding national obesity rates.^25^

**TABLE 1:** Published epidemiological data on idiopathic intracranial hypertension.

	Mean age (years)	Female‐to‐male ratio	Total incidence (per 100,000 people per year)	Female incidence (per 100,000 people per year)
Libya^9^ (1983–1984)	30.2	All patients (*n* = 23) were females	1.7	3.6
Iowa^10^ (1984–1985)	26.7	8:1	0.9	3.5^a^
Louisiana^10^ (1984–1985)	28	4.3:1	1.07	‐
Benghazi, Libya^11^ (1982–1989)	28	15.2:1	3.2	5.9
Rochester^12^ (1976–1990)	27.8	8:1	0.9	1.6
Northern Ireland^13^ (1991–1995)	29	5.7:1	0.5	0.9
Israel^14^ (1998–1999)	32.3	14:1	0.94	1.82
Sheffield^15^ (2007–2008)	28.1	15:1	1.56	2.86
Sweden^16^ (2006–2013)	31.0 (Females) 42.9 (Males)	6.1:1	0.65	1.1
Northern Ireland^17^ (2007–2014)	29.4	44:1	2.36	4.65
USA^18^ (1997–2016)	‐	5.47:1	1.15	1.97
Wales^19^ (2017)	30.1	5.7:1	7.8	‐
Kuwait^20^ (2018)	32.1	9.6:1	1.6	‐

^a^ Females aged 15–44 years.

In addition to obesity, research by Brahma et al. indicates a distinct correlation between race/ethnicity and IIH.^26^ This was further supported by other research findings that indicate more severe symptoms in black patients with IIH, supporting the notion of racial differences in IIH.^27^ The interaction between race/ethnicity, obesity, and IIH is further complicated by complex socioeconomic health determinants.^26^ Specifically, areas with low‐income populations and limited access to food are associated with an increased risk of obesity, disproportionately affecting minority communities.^28^, ^29^, ^30^ With this increasing incidence, the financial burden on healthcare providers has also increased, gradually drawing more attention to this issue.

## 3 RISK FACTORS

### 3.1 Obesity/weight gain

**FIGURE 1:**
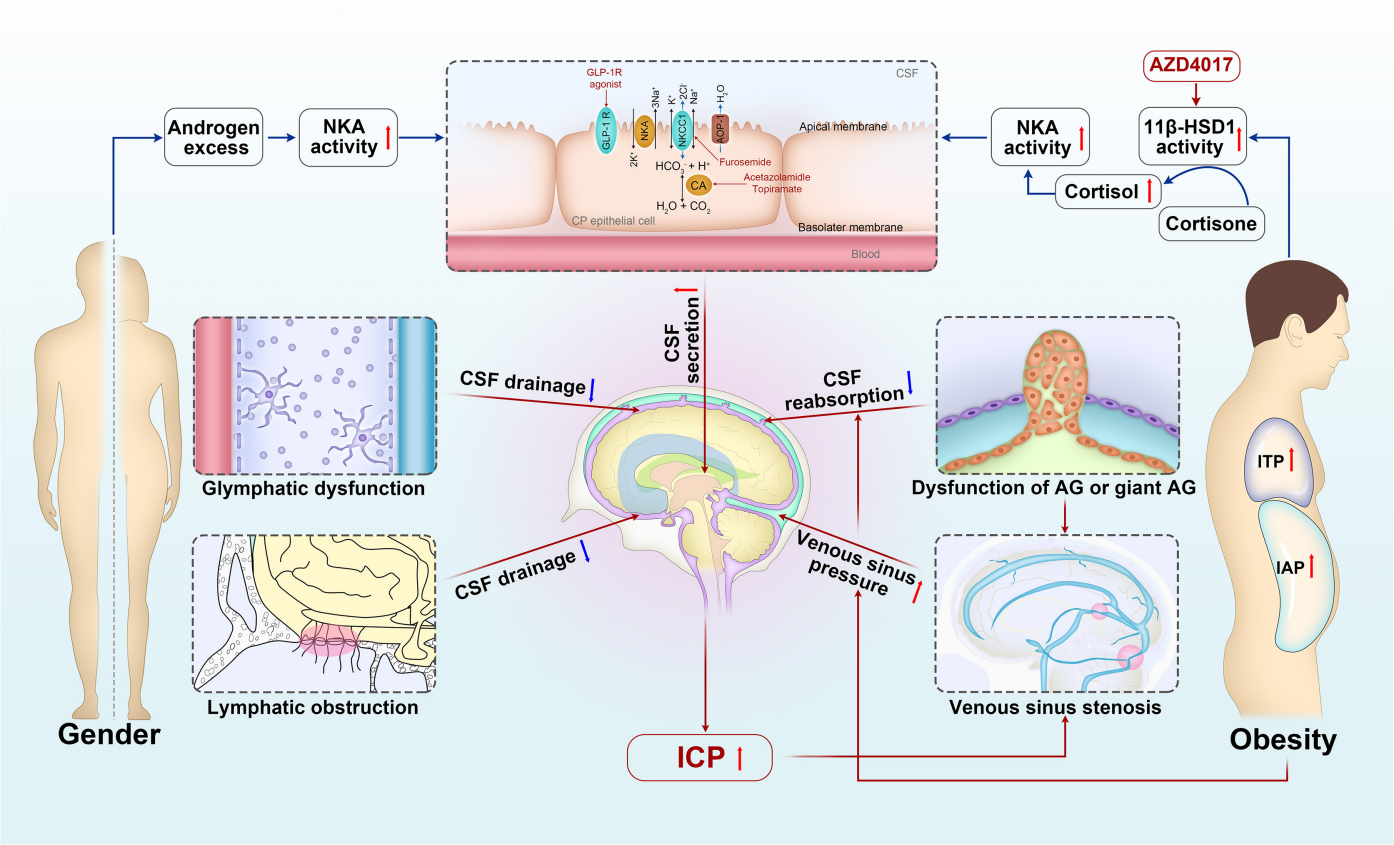
Proposed pathophysiological mechanisms underlying IIH and the target sites of major therapeutic agents. The pathogenesis of IIH is multifaceted, with no single explanation; key mechanisms include heightened CSF secretion from the CP, decreased CSF reabsorption through AG, venous sinus stenosis leading to venous hypertension, and dysfunction within the lymphatic and glymphatic systems. Additionally, adipose tissue dysfunction and hormonal disruption in IIH play an important role in promoting CSF secretion. Both acetazolamide and topiramate work by inhibiting carbonic anhydrase activity. AZD4017 acts as an inhibitor of 11β‐HSD1, thereby diminishing the local concentration of cortisol. GLP‐1R agonists attach to and stimulate the GLP‐1R, leading to the suppression of NKA activity and a decrease in sodium excretion. 11β‐HSD1, 11β‐hydroxysteroid dehydrogenase type 1; AG, arachnoid granulation; AQP‐1, aquaporin‐1; CA, carbonic anhydrase; CP, choroid plexus; CSF, cerebrospinal fluid; GLP‐1R, glucagon‐like peptide‐1 receptor; IAP, intra‐abdominal pressure; ICP, intracranial pressure; IIH, idiopathic intracranial hypertension; ITP, intrathoracic pressure; NKA, Na^+^/K^+^‐ATPase; NKCC1, Na^+^‐K^+^‐2Cl ^−^ cotransporter.

Obesity and recent weight gain are the main risk factors for IIH and are closely associated with its onset and recurrence.^2^, ^31^ Although the precise role of obesity in IIH is not yet fully understood, the prevailing theories suggest that central obesity may lead to elevated intra‐abdominal pressure, resulting in increased pressure in both the pleural space and heart, thus obstructing venous return from the brain and playing a key role in the onset and recurrence of IIH (Figure 1).^32^ A matched case‐control study by Daniels et al., which enrolled 34 female patients with newly diagnosed IIH and 41 patients with other neuro‐ophthalmological disorders, indicated that higher weight gain and body mass index (BMI) were associated with an increased risk of IIH.^33^ The study also found that even patients without obesity (BMI < 30 kg/m^2^) were at a higher risk of IIH in cases of moderate weight gain.^33^ In a retrospective study by Ko et al., 50 females diagnosed with IIH were included.^31^ The study showed that patients who experienced IIH recurrence had experienced significant increases in BMI, as compared to those without recurrence in the same cohort.^31^ Obesity is also a risk factor for adverse visual outcomes in patients with IIH. Patients with a higher BMI often exhibit more severe papilledema and have an increased risk of vision loss.^34^, ^35^, ^36^ Similarly, a study involving 25 patients overweight with IIH who followed a low‐energy diet revealed significant reductions in ICP, improvements in symptoms, and decreased swelling of the optic disc after 3 months.^37^

### 3.2 Heredity

Familial IIH (FIIH) was first reported in 1969.^38^ In the Idiopathic Intracranial Hypertension Treatment Trial (IIHTT), 5% of the patients had a family history of IIH, suggesting that genetic factors may increase the risk of developing this condition.^21^ Although various genetic theories have been proposed, their mode of inheritance remains unclear. An analysis by Qiao et al. that included 39 FIIH pedigrees indicated that parent–child transmission occurred in nearly 50% of cases, whereas about one‐third of siblings were affected.^39^ Some studies have not found any genetic variations strongly associated with IIH.^40^, ^41^ Therefore, the roles of genetic and epigenetic factors in IIH need further exploration.

### 3.3 Drugs

The use or discontinuation of certain drugs is associated with the onset or recurrence of IIH.^42^, ^43^, ^44^ A comprehensive systematic review that examined 259 incidents of medication‐related IIH used the Koh algorithm to assess the strength of the association and risk categorization and revealed that vitamin A‐derivatives and tetracycline antibiotics had the strongest association with IIH, while corticosteroids were moderately linked and oral contraceptives exhibit a weaker connection.^42^

All‐trans retinoic acid (ATRA), an active derivative of vitamin A, dose‐dependently stimulates expression of the gene encoding aquaporin‐1 (AQP‐1) in erythrocytes.^45^ AQP‐1 is also prominently expressed in the apical membranes of choroid plexus (CP) epithelial cells and contributes significantly to CSF production.^46^ The transformation of vitamin A into ATRA, leading to increased levels of AQP‐1 within the epithelium of the CP, may explain the elevated ICP observed with excessive or prolonged consumption of vitamin A‐derivatives.^47^ However, this theory remains controversial. Libie et al. conducted a prospective randomized controlled trial to measure vitamin A metabolite levels in the serum and CSF of patients with IIH.^47^ The results indicated a negative correlation between BMI and serum ATRA levels (*r* = −0.25, *p* = 0.0005), while no significant correlation was found with serum retinol (*r* = −0.14, *p* = 0.17). Interestingly, CSF pressure was only slightly negatively correlated with ATRA levels in the CSF (*r* = −0.27, *p* = 0.008). These findings support the results of an animal study that indicated that the increase in AQP‐1 in the CP of obese rats with an increase in ICP appears to be independent of retinol metabolism.^48^

A retrospective cohort study that included 52 cases of tetracycline‐induced pseudotumor cerebri (PTC‐T) and 302 patients with IIH revealed that the PTC‐T group was characterized by a younger mean age (19.8 vs. 28.1 years, *p* < 0.001), a significantly lower obesity rate (43.8% vs. 79.2%, *p* < 0.001), and a reduced rate of recurrence (4.0% vs. 16.5%, *p* < 0.001) than were those with IIH.^49^ Eldweik et al. conducted a retrospective examination of 728,811 patients from the US Clinformatics Data Mart database. They showed that cycline antibiotics increased the risk of PTC syndrome or papilledema (doxycycline: hazard ratio [HR], 1.70; 95% confidence interval [CI], 0.98–2.97; minocycline: HR, 1.91; 95% CI, 1.11–3.29). However, after adjusting for confounding factors, no statistically significant association was found (*p* = 0.06 for doxycycline; *p* = 0.08 for minocycline).^50^

The withdrawal of corticosteroids has been associated with the development of IIH.^51^, ^52^ In 1966, it was hypothesized that patients with PTC could exhibit impaired synthesis or release of endogenous adrenocorticotropic hormone (ACTH).^53^ Johnston proposed that acute cessation of steroids could lead to prolonged suppression of pituitary ACTH reserves, thus increasing resistance to CSF absorption.^54^ The prevailing theory on the regulation of CSF mechanisms by steroids suggests that cortisol activates mineralocorticoid receptors, which in turn increases gene transcription of epithelial sodium channels and Na^+^/K^+^‐ATPase (NKA).^55^ Furthermore, studies have observed an elevated activity of 5α‐reductase in patients with IIH, a key enzyme in cortisol metabolism and testosterone conversion to dihydrotestosterone.^56^ Previous research by O'Reilly et al. also reported significantly elevated testosterone levels in the CSF of female patients with IIH, indicating a potential regulatory role for androgens in CSF secretion dynamics.^57^

The correlation between oral contraceptive pills (OCPs) and IIH has long been the subject of debate. However, recent studies have increasingly indicated that OCPs may not constitute a risk factor for IIH.^58^, ^59^ Kilgore et al. conducted a retrospective case‐control study that confirmed no significant association between the use of OCPs and IIH (odds ratio [OR], 0.55, *p* = 0.146).^58^

### 3.4 Obstructive sleep apnea

Obstructive sleep apnea (OSA) is closely associated with ICP.^60^ The possible mechanisms through which OSA leads to increased ICP include: 1) hypoxia and hypercapnia resulting in cerebral vasodilation and increased cerebral blood flow, which subsequently elevate arterial and central venous pressure; 2) central obesity causing an increase in abdominal pressure, thereby affecting cerebral venous return; and 3) factors such as neck obesity, upper airway constriction, mouth breathing, or a forward head posture, all of which contribute to increased jugular vein resistance. OSA is more common in male patients with IIH.^61^, ^62^ However, its role as an independent risk factor for IIH remains debatable. In a substudy of the Idiopathic Intracranial Hypertension Weight Trial (IIH:WT), Yiangou et al. included 46 patients with IIH, 19 of whom had OSA. After adjusting for BMI as a confounding factor (*R* ^2^ = 0.522, *p* = 0.017), they observed a significant positive correlation between the improvement in the apnea–hypopnea index (AHI) and changes in papilledema (*r* = 0.543, *p* = 0.045), although the correlation between changes in AHI and a decrease in ICP was not significant. However, OSA appears to be associated with adverse visual outcomes in IIH, possibly due to intermittent hypoxia that exacerbates optic nerve ischemia.^27^, ^63^ A case of a patient with malignant IIH was reported that relapsed 8 months after lumboperitoneal ventricular shunt surgery, despite treatment with drugs, such as acetazolamide and topiramate, and repeated lumbar punctures. Subsequent diagnosis of concurrent OSA followed by tonsillectomy led to marked improvements in both conditions.^64^ This highlights the potential of OSA treatment to improve the long‐term prognosis of patients with IIH. Most risk factors require additional empirical validation to quantify their relationship with IIH and to devise appropriate preventive measures for modifiable risk factors.

## 4 HYPOTHESIS OF PATHOLOGICAL MECHANISM

The etiology of IIH is multifactorial and is not yet fully elucidated.^65^ Current research efforts have focused on investigating the potential causes, including disruptions in CSF dynamics, elevated venous pressure, systemic metabolic imbalances, and inflammatory processes (Figure 1).^22^, ^66^

### 4.1 Impaired CSF homeostasis

#### 4.1.1 Increased secretion of CSF

CSF production by the CP is a complex process involving multiple ion channel transporters, including NKA, Na^+^‐K^+^‐2Cl^−^ cotransporter (NKCC1), and aquaporins.^67^ Recent hypotheses suggest that excessive levels of androgens, adipokines, and glucocorticoids may contribute to enhanced CSF secretion in IIH, potentially by upregulating ion channels and AQP activity.^68^ NKCC1 is a bidirectional transporter protein that predominantly operates through phosphorylation‐dependent mechanisms. Although the precise transport direction of NKCC1 has yet to be elucidated, it is suspected to be influenced by ion concentrations on either side of the CP.^69^ The SPAK‐NKCC1 co‐transporter complex plays an important role in the excessive secretion of CSF during post‐hemorrhagic hydrocephalus.^70^ Studies have shown that genetic knockout of SPAK or pharmacological inhibition of NKCC1 activity can restore normal CSF secretion in rats.^70^, ^71^, ^72^ Wardman et al. simulated the physiological characteristics of obesity and excessive androgen, commonly observed in patients with IIH, using female rats fed a high‐fat diet (HFD) supplemented with testosterone.^73^ These findings indicate that elevation of ICP in rats with HFD was not due to an increase in CSF secretion but that rats treated with testosterone showed an increase in CSF secretion rate and NKCC1 activity. Therefore, androgens could cause IIH by stimulating NKCC1 activity, augmenting CSF secretion.^73^ Aquaporins mediate the exchange of intracranial intracellular fluid, interstitial fluid, CSF, and blood.^74^ AQP‐1 is predominantly located in the apical membrane of CP epithelial cells.^75^ Studies, such as those by Uldall et al., have observed elevated expression of AQP‐1 in the CP of obese rats.^48^ Similarly, Oshio et al. reported a decrease in ICP and CSF production in mice with AQP1 knockout.^76^ These findings suggest that AQP‐1 plays a role in ICP regulation by influencing CSF secretion.^48^, ^76^ In conventional IIH treatment, acetazolamide, which is mainly known for its NKA inhibitory effect, is used to reduce ICP and can suppress the expression of AQP‐1.^77^ Recent studies have provided evidence that increased CSF production may contribute to the incidence of IIH. However, more research is required to elucidate the precise mechanisms underlying the relationship between CSF production and IIH.

#### 4.1.2 Obstruction of the venous drainage pathway

##### Dysfunction of arachnoid granulation

Arachnoid granulations (AGs) are projections of the arachnoid membrane that extend to the dural venous sinus and are commonly located in the transverse, sigmoid, and superior sagittal sinuses.^78^ They range in diameter from 0.5 to 1.5 cm, and their quantity increases with age. According to the traditional theory of CSF circulation, once the CSF enters the subarachnoid space, it returns to the venous system through AGs, a process driven by the hydrostatic pressure gradient between the CSF and dural veins.^79^ With the increase in the CSF pressure in the subarachnoid space, both the quantity and size of AGs appear to increase, which can lead to an increase in the venous sinus pressure due to giant AGs.^80^, ^81^ A retrospective investigation involving 65 patients with IIH and 144 controls demonstrated a significant disparity in the AG count for female patients aged 20–45 years as compared with the control group (*p* = 0.04).^82^ Furthermore, in patients with IIH, the presence of AGs is negatively correlated with magnetic resonance imaging (MRI) findings, such as empty sella syndrome.^82^ Individuals with little or no AGs may have limited ability to compensate for increased ICP through augmented CSF absorption through AGs, which could make them more susceptible to developing IIH.^82^ Insufficient AG compensation may contribute to the incidence and progression of IIH.^82^ Durst et al. found that the proportion of AG was significantly elevated in individuals with unilateral transverse sinus stenosis as compared to those without this condition (70% vs. 18%).^83^ A study by Lublinsky et al. revealed that AG counts were higher in IIH patients than in healthy individuals; however, this difference did not reach statistical significance (2.41 ± 1.12 vs. 1.50 ± 0.54, *p* = 0.096).^81^ Recent studies have suggested that AGs may not be as efficient as mature structures, which may represent fibrotic degeneration of the arachnoid villi.^84^ Furthermore, many individuals with normal CSF systems lack AGs, suggesting that their actual role in CSF drainage may be less significant than previously believed.^85^, ^86^ The pathogenic mechanism underlying AG formation remains unclear: whether it is due to obstructed compensatory functions in promoting CSF drainage or whether it leads to venous sinus stenosis, thus exacerbating ICP elevation, needs to be determined.

##### Elevated venous pressure caused by venous sinus stenosis

Patients with IIH often have intracranial venous sinus stenosis, which is most frequently observed in the transverse sinus or at its junction with the sigmoid sinus.^87^ Bilateral transverse sinus stenosis constitutes 23%–93% of IIH cases, in significant contrast to the prevalence of 5%–7% observed in the general population.^83^, ^87^, ^88^ Retrograde venous angiography reveals that venous sinus stenosis increases venous sinus pressure, and research has indicated a strong correlation between CSF opening pressure and venous sinus pressure.^89^, ^90^ In the past two decades, a persistent debate has focused on whether venous sinus stenosis is a causative factor of IIH or simply a consequence of elevated ICP levels. Buell et al. documented a case in which a significant volume of CSF was rapidly removed by lumbar puncture, effectively reducing ICP and the severity of venous sinus stenosis.^91^ Subsequently, this case study was considered support for the concept that venous sinus stenosis is a secondary manifestation of IIH.^91^ Additionally, the existence of a positive feedback loop and the “self‐sustaining venous collapse” theoretical model have been proposed, suggesting the possibility of a bidirectional causal relationship between venous sinus stenosis and IIH.^92^ Factors such as weight gain or hormonal changes may initiate mild elevations in ICP in populations prone to these changes.^92^, ^93^, ^94^, ^95^ Currently, two forms of venous sinus stenosis are recognized in relation to IIH. First, intrinsic stenosis, characterized by abnormal internal anatomical structures in the venous sinus lumen (such as swollen AG, fibrous membranes, etc.^88^), typically presenting as discontinuous focal stenosis, and second, extrinsic stenosis, arising from factors that increase ICP, which in turn compresses the venous sinus, reduces venous sinus compliance, and causes sinus wall collapse, generally presenting as segmentally smooth stenosis.^96^ Patients may have features of endogenous and exogenous stenoses, and effective identification of these types of stenoses could contribute to the creation of tailored treatment strategies.^97^

#### 4.1.3 Glymphatic and lymphatic systems

Due to advances in molecular biology and imaging technology, assessment of the lymphatic drainage pathways of CSF have improved significantly, and these pathways have been shown to involve routes such as passing through the cribriform plate adjacent to the cranial nerve sheath, ultimately reaching the nasal mucosa, and extending through the meningeal lymphatic vessels (mLVs) to the deep cervical lymph nodes.^98^ Alperin et al. observed that the extra‐ventricular CSF space in an IIH group was approximately 30% larger (220 mL) than that in the control group, presumably due to congestion of the glymphatic system.^99^ Recently, Lenck et al. proposed that impairment of interstitial fluid transport from the glymphatic system to the venous blood could trigger the hydrodynamic cascade of IIH.^84^ A primary restriction in the venous pathway for CSF outflow results in secondary congestion within the glymphatic system and overflow of the lymphatic CSF outflow pathway, potentially leading to the clinical manifestations of IIH.^84^ Furthermore, this microscopic impairment of the venodural junction may be responsible for the development of venous sinus stenosis, particularly in cases of transverse sinus stenosis, which may be a direct consequence of glymphatic system overload.^100^ Eide et al. used the contrast agent gadobutrol as a CSF tracer, and consecutive, standardized T1 MRI scans were acquired over 48 h after intrathecal tracer administration in 15 patients with IIH and 15 controls. The results showed that the extravascular distribution of the CSF tracer increased and clearance was delayed in multiple brain regions, including the frontal, temporal, cingulate, insular, cerebellar, and brainstem regions, providing in vivo evidence of impaired glymphatic function in IIH.^101^ Based on previous research on ultrastructural analyses of cortical brain biopsies obtained from frontal gray matter in patients with IIH, Eide et al. suggested that structural alterations and impaired metabolism at the glia–neurovascular interface may be the mechanisms underlying glymphatic dysfunction, and explored this in more depth.^102^ In subsequent studies, they found that, of 13 patients with IIH, 12 showed abnormal structural changes at the glia–neurovascular interface, as evidenced by a compensatory increase in perivascular AQP‐4 expression, accompanied by extensive pathological changes, including patchy astrogliosis proliferation, increased pathological mitochondria, and shortened post‐synaptic density length.^103^, ^104^ Additionally, a highly significant positive correlation was found between the degree of blood–brain barrier leakage and astrocyte proliferation in IIH.^105^ Despite the discovery of a link between the function of the glymphatic system and various central nervous system disorders, particularly degenerative conditions such as Alzheimer's disease and idiopathic normal‐pressure hydrocephalus, few studies have explored the impact of the glymphatic system on CSF circulation and ICP regulation. Therefore, further studies are needed to investigate the mechanistic link between the glymphatic system and IIH, which will aid in the discovery of new biomarker‐based diagnostic targets and may provide new ideas for treatment.

### 4.2 Systemic metabolic dysregulation

The presence of central adiposity has been shown to play a central role in the development of metabolic manifestations, morbidity, and overall mortality.^106^, ^107^ To advance our understanding of the role of adipocytes in IIH, Westgate et al. examined the clinical characteristics and cell phenotypes of omental and subcutaneous adipose tissue of individuals matched in age, sex, and BMI, with and without IIH.^108^ Histomorphometric analysis revealed that omental adipocytes in patients with IIH were smaller and present at a higher density per area than they were in control subjects.^108^ Furthermore, RNA sequencing analysis has shown that adipose tissue in individuals with IIH is transcriptionally primed for lipogenesis and increased calorie intake.^108^ Consequently, different characteristics were observed in both types of tissue, suggesting that adipose tissue in IIH is predisposed to lipid accumulation, which could lead to increased adipose mass in affected individuals.^108^ The observation of truncal adiposity in individuals with IIH led to the hypothesis that IIH is a metabolic syndrome characterized by insulin resistance.^108^ Westgate et al. tested this hypothesis and found increased insulin and leptin resistance in patients with IIH as compared to sex‐matched and BMI‐matched controls, further supporting the notion of metabolic dysregulation in IIH.^108^

A hormone metabolome study using liquid chromatography‐mass spectrometry (LC‐MS) revealed a distinct androgen profile in patients with IIH, characterized by elevated levels of CSF and testosterone as compared to individuals with simple obesity or lean controls.^57^ Additionally, an increase in the activity of 5α‐reductase, an enzyme essential to androgen and glucocorticoid metabolism, was observed in patients with IIH.^57^ Testosterone increases CSF secretion in vitro, while chronic testosterone treatment in rats also leads to an increased CSF secretion rate in vivo, further supporting the role of androgens in IIH pathogenesis.^73^ Consistent with the hypothesis that excess testosterone contributes to the pathophysiology of IIH, a growing body of evidence has described the development of elevated ICP, similar to IIH, in transgender individuals undergoing a female‐to‐male transition with testosterone therapy.^109^, ^110^ Glucocorticoid dysregulation has also been implicated in the development of IIH. A study has revealed elevated activities of 5α‐reductase and 11β‐HSD1 in urine samples of IIH patients as compared to age‐, sex‐, and BMI‐matched controls.^56^ Additionally, increased 11β‐HSD1 activity has been observed in adipose tissue from those with IIH.^56^ This enzyme, which converts inactive cortisone into active cortisol, is present in epithelial cells of the CP and influences cortisol levels in the CSF.^111^ Furthermore, the administration of exogenous glucocorticoids has been shown to augment CSF secretion in rodent models.^112^ Therefore, glucocorticoids and their metabolizing enzymes may play a significant role in modulating CSF dynamics in patients with IIH. In particular, weight loss achieved by bariatric surgery or a 3‐month diet regimen was associated with reduced 11β‐HSD1 activity in patients with IIH, correlated with a significant decrease in ICP.^56^, ^113^

An untargeted case‐control metabolomic study involving a cohort of 84 individuals with active IIH identified an altered CSF‐to‐serum urea gradient, which was significantly correlated with the severity of headache.^114^ Additionally, the study revealed that levels of acetate, a precursor of adenosine, were significantly elevated in the baseline CSF of patients with IIH as compared to that of healthy controls. These elevated levels correlated with Headache Impact Test‐6 scores and increased headache severity, suggesting that higher concentrations of acetate exacerbate headache.^114^ Furthermore, a notable reduction in acetate production was observed. Similarly, Alimajstorovic et al. investigated the global metabolome of individuals with IIH by using a discovery‐based approach and ultra‐high‐performance LC–MS.^115^ This study found that the concentrations of acylpyruvates, including formylpyruvate and maleylpyruvate–fumarylpyruvate, were lower in the CSF and higher in the serum of patients with IIH than in BMI‐ and sex‐matched individuals without IIH.^115^ Pathway enrichment analysis revealed perturbations in lipid metabolite pathways and amino acid metabolism, specifically those involving arginine, proline, and histidine.^115^ Furthermore, alterations in metabolic pathways were associated with IIH disease characteristics, such as visual function and improved with IIH treatment. Reducing ICP does not change metabolic profiles, underscoring the multifaceted nature of IIH pathophysiology. Therefore, the management of IIH as a metabolic disease is essential, as it could potentially decrease disease severity. More research is imperative to determine whether targeted interventions to correct metabolic disorders can effectively reduce ICP in patients with IIH.

### 4.3 Inflammatory activation

Given that obesity is characterized by a persistent, low‐grade inflammatory state marked by the systemic presence of various cytokines, research has been conducted to explore whether inflammatory pathways that cannot be solely attributed to obesity contribute to the development of IIH.^116^ Samancı et al. analyzed serum samples of 36 patients with IIH and of 40 healthy controls and discovered that interleukin‐1β levels were elevated (*p* = 0.012) in the IIH group, while interleukin‐8 and tumor necrosis factor‐α (TNF‐α) levels were reduced (*p* < 0.001 and 0.008, respectively), suggesting a unique inflammatory pattern in IIH.^117^ In contrast, Fahmy et al. concluded that serum TNF‐α levels were significantly elevated in patients with IIH (*p* < 0.001), presenting a negative correlation with the grade of perimetry (R = −0.36, *p* = 0.02).^118^ Genizi et al. conducted a prospective cross‐sectional study in three Israeli centers, using enzyme‐linked immunosorbent assays to detect cytokine and chemokine levels in CSF samples from 60 children aged 0.5–18 years. The study revealed a significant increase in chemokine ligand (C–C motif) 2 levels in the PTC group as compared to the control group (*p* < 0.05).^119^ Despite the variability in the results in different studies, the consistently observed abnormal expression of inflammatory mediators in most of them highlights a potential link between inflammation and IIH.^117^, ^120^, ^121^, ^122^, ^123^ However, it should be noted that these investigations are constrained by factors such as limited sample sizes and heterogeneous populations, necessitating a cautious interpretation of these findings and highlighting the need for more extensive research.

## 5 CLINICAL SPECTRUM OF IIH

Headache is the most reported symptom, affecting 75%–94% of patients with IIH. Almost all cases present with papilledema, which commonly results in varying degrees of visual impairment.^5^, ^21^ Other symptoms include pulsatile tinnitus, nausea, vomiting, and neck and back pain. With a more profound understanding of the disease, cognitive impairment is now recognized as a manifestation of IIH.

### 5.1 Headache

Headaches in IIH are heterogeneous, manifest as intermittent or persistent, and resemble pulsating or pressure‐like pain. They often worsen with coughing or during the Valsalva maneuver.^124^, ^125^ Headaches can be located in the frontal lobe, retroorbital region, or involve the entire head.^124^ Up to 45% of patients with IIH have a history of migraine, and up to 80% of patients with headaches show a migraine phenotype.^126^ The link between IIH and migraine continues to be a topic of debate. A multicenter randomized controlled trial (IIH:WT) involving 66 patients with IIH showed that the frequency and severity of headache correlated positively with ICP.^127^ However, some patients complain of chronic migraine‐like headaches despite resolution of papilledema or normalization of ICP.^128^, ^129^ It should be mentioned that IIH without papilledema (IIHWOP) is increasingly recognized as a contributing factor to refractory headaches. Improving the diagnosis of IIHWOP could help to reduce the prevalence of headaches; however, challenges persist in its accurate diagnosis.^130^

### 5.2 Visual symptoms

Papilledema typically affects both eyes symmetrically in IIH; however, instances of highly asymmetric and rare unilateral papilledema have been reported, affecting 3.6%–10% and 1.4% of patients, respectively.^131^ The gold standard for interpreting papilledema is the Frisén grading scale, which was introduced in 1982.^132^ Transient visual obscuration is prevalent, while a minority of patients with fulminant IIH face the risk of permanent vision loss.^133^ The risk of permanent vision loss is directly correlated with the severity of papilledema. Therefore, the prompt identification of and intervention for severe papilledema are crucial.^134^ Compared to typical patients with papilledema, patients with IIHWOP generally experience more photopsia, less diplopia and blind spot enlargement, and a lower risk of vision loss.^135^ Horizontal diplopia accounts for <20% of cases and is primarily caused by unilateral or bilateral abducens nerve palsy, with additional contributing factors such as cranial neuropathy.^21^ All patients suspected of having IIH must undergo a thorough ophthalmic assessment, including a visual field test, fundus examination, and optical coherence tomography. In cases of papilledema uncertainty, additional diagnostic tools, such as ocular ultrasound and fluorescein angiography, should be employed.

### 5.3 Cognitive impairment

Multiple studies have confirmed the presence of cognitive impairment in individuals with IIH.^136^, ^137^, ^138^ A prospective case‐control study in Denmark assessed the cognitive function of 31 patients with IIH and of 31 healthy individuals. Patients with IIH performed significantly worse in four of the six cognitive domains (*p* < 0.02), indicating some form of multidomain cognitive impairment in patients with IIH.^139^ Furthermore, Grech et al. identified deficiencies in multiple cognitive domains in a study involving 66 female patients with IIH and corroborated that these cognitive deficits could be improved by reducing ICP.^138^

## 6 TREATMENT

**FIGURE 2:**
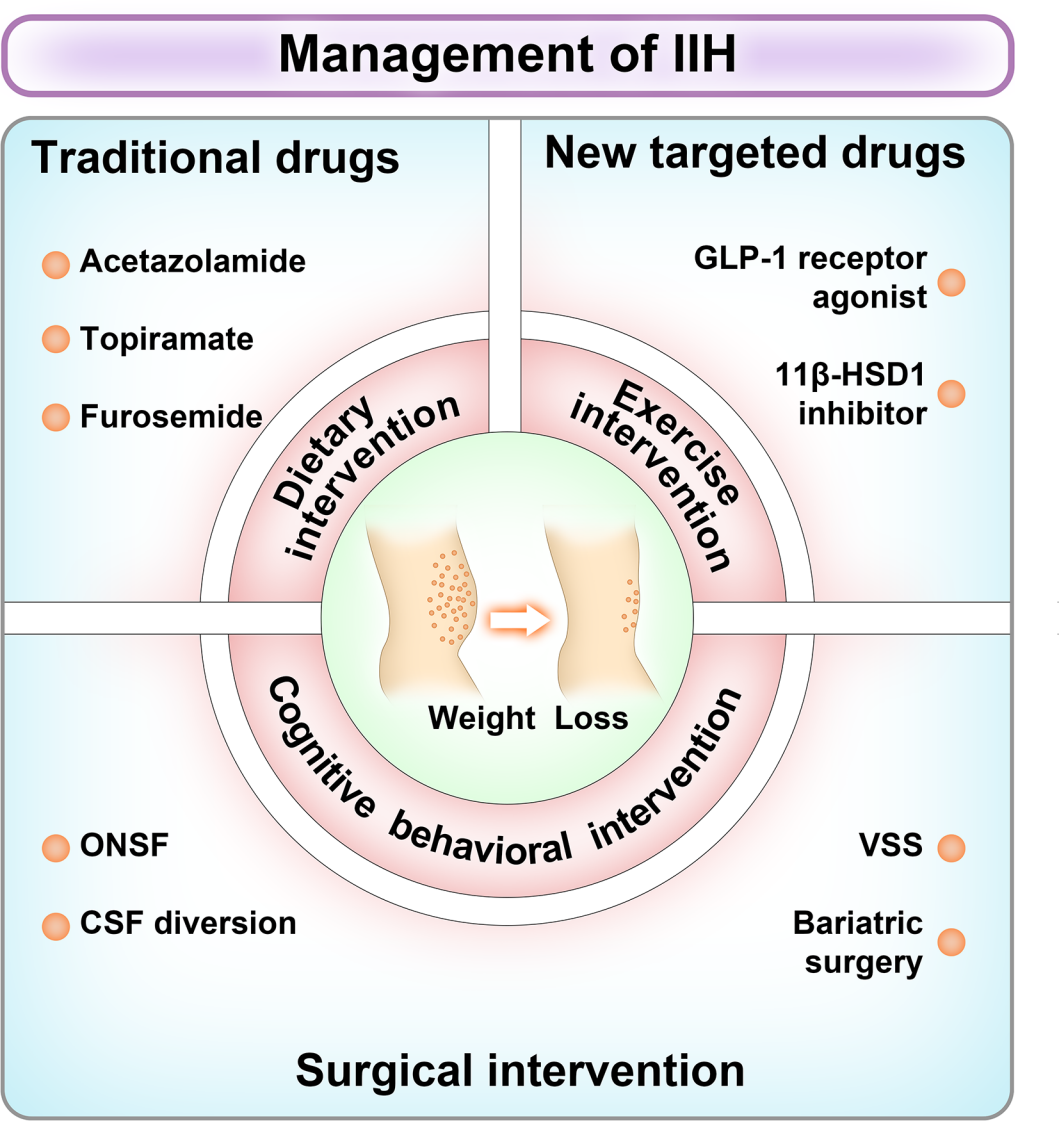
Management of IIH. The treatment of IIH focuses on vision preservation and headache relief. Effective weight loss and long‐term weight control are the core of IIH management. Overweight or obese patients should adopt a scientific and healthy lifestyle intervention for weight loss, including diet, exercise and behavioral intervention. 11β‐HSD1, 11β‐hydroxysteroid dehydrogenase type 1; CSF, cerebrospinal fluid; GLP‐1, glucagon‐like peptide‐1; IIH, idiopathic intracranial hypertension; ONSF, optic nerve sheath fenestration; VSS, venous sinus stenting.

The core principles of IIH management include immediately addressing the underlying disease at diagnosis, protecting vision, and minimizing the frequency of headaches.^6^ Typically, a combination of weight loss and medication therapy is used, along with surgical interventions that can be considered after comprehensively assessing the patient's clinical profiles and associated risks (Figure 2).

### 6.1 Weight management

Persistent weight loss is a key strategy for mitigating the symptoms of intracranial hypertension and reducing the risk of complications associated with obesity.^140^, ^141^, ^142^ Although the 2018 consensus guidelines on the management of IIH suggested that all patients with a BMI >30 kg/m^2^ should prioritize weight management, they did not specify the amount of weight loss necessary to improve the disease or the most effective weight loss strategy.^6^ The IIH:WT, involving 66 females with active IIH and a BMI of ≥35 kg/m^2^ at five UK hospitals, showed that bariatric surgery was significantly more effective in reducing ICP than were community weight management programs.^143^ A sub‐study of IIH:WT mice further assessed the necessary weight loss to alleviate ICP and revealed that loss of 24% body weight was related to disease remission.^140^ A recent systematic review, which included 17 studies, evaluated the effects of various weight loss interventions on the reduction of ICP, as well as their impact on vision, headache outcomes, and quality of life.^141^ Bariatric surgery led to the highest mean reduction in ICP (−11.9 cmH_2_O), closely followed by a lifestyle intervention combined with acetazolamide (−11.2 cmH_2_O). A very‐low‐energy diet intervention (−8.0 cmH_2_O) and a lifestyle intervention (−5.2 cmH_2_O) resulted in smaller reductions.^141^ Bariatric surgery involves gastric banding, gastric bypass, and sleeve gastrectomy.^115^ In contrast, gastric bypass achieved a greater, faster, and sustained reduction in ICP than did the other two methods.^115^, ^140^, ^141^

### 6.2 Medical treatment

#### 6.2.1 Traditional drugs

Medications frequently prescribed for IIH include diuretics, such as acetazolamide and furosemide, anticonvulsants, such as topiramate, and other agents, such as octreotide.^6^ Among these, acetazolamide is used as the first‐line treatment.^144^ It is a carbonic anhydrase inhibitor that interferes with CSF production. In the IIH treatment trial, the largest randomized controlled trial to date, the perimetric mean deviation (1.43 vs. 0.71 dB, *p* = 0.05) and papilledema grade (−1.31 vs. −0.61, *p* < 0.001) showed improvement after 6 months in patients treated with a combination of acetazolamide and a low‐sodium diet, as compared to those on a diet alone.^144^ However, acetazolamide‐induced side effects are prevalent and include sensory disorders, loss of appetite, oral metallic taste, fatigue, nausea, vomiting, electrolyte imbalances, mild metabolic acidosis, and kidney stones.^145^ Ball et al. reported a withdrawal rate of 48% from treatment due to adverse reactions at an average dose of 1.5 g of acetazolamide.^146^ A Cochrane systematic review conducted in 2015 also noted insufficient evidence to determine the efficacy of acetazolamide.^147^

Topiramate is the primary therapy for migraine prevention and is commonly used as a substitute for acetazolamide. This substitute may offer greater relief for patients with IIH who experience migraine‐like headaches. Tests on healthy rats showed that topiramate outperformed acetazolamide in reducing ICP.^148^ Nevertheless, placebo‐controlled studies are required to assess the efficacy of topiramate. The effectiveness of diuretics, such as furosemide and amiloride, in IIH treatment remains uncertain due to limited research and insufficient clinical evidence.

#### 6.2.2 New targeted drugs

Recently, the development of new targeted medications has become a major research focus. Currently, the most promising medications for treating IIH are 11β‐HSD1 inhibitors and glucagon‐like peptide‐1 receptor agonists (GLP‐1RAs). 11β‐HSD, which comes in two variants, 11β‐HSD1 and 11β‐HSD2, plays a crucial role in controlling glucocorticoid levels in the body.^149^ Cortisol is broken down into inert metabolites by 11β‐HSD2 in the kidneys. These inactive metabolites are then regenerated into cortisol through 11β‐HSD1 in tissues such as the liver and fat.^149^ In individuals with obesity, 11β‐HSD1 activity is increased in subcutaneous adipose tissue, and this activity correlates positively with BMI, as indicated by increased messenger RNA levels of 11β‐HSD1 (*p* < 0.05).^150^ 11β‐HSD1 expression is observed not only in the kidneys, liver, and adipose tissue but also in the CP, which could involve it in CSF regulation.^111^ Sinclair et al. found that a reduction in 11β‐HSD1 activity correlated with weight loss (15.2 ± 7.8% of body weight), which was significantly associated with a decrease in ICP (*r* = 0.504; *p* = 0.028).^113^ AZD4017 is a reversible competitive inhibitor of 11β‐HSD1.^151^ A UK‐based Phase II multicenter, randomized controlled trial assessed the therapeutic efficacy and safety of AZD4017 for treating IIH, as compared to a placebo. The study revealed that AZD4017 was well‐tolerated and significantly reduced ICP by 4.3 cm CSF over a 12‐week period, outperforming the 0.3 cm decrease in CSF in the placebo group.^152^ Subsequently, the research group conducted a single‐center, randomized controlled trial to explore the broader effects of AZD4017 on metabolic profiles. These findings revealed that patients treated with AZD4017 experienced improvements in their lipid levels, mainly characterized by decreased cholesterol and increased high‐density lipoproteins.^8^

GLP‐1, a neuropeptide secreted primarily in the intestine, plays multiple roles in the body.^153^ It is not only extensively spread in pancreatic cells and in the gastrointestinal tract but is also expressed in the central nervous system, such as the choroid.^154^, ^155^ GLP‐1RAs not only lower blood sugar, but also boost satiety and reduce food intake by engaging the hypothalamus, ultimately resulting in weight loss.^156^ A study showed that GLP‐1RAs could effectively lower ICP in normal and hydrocephalus rat models by minimizing NKA activity.^157^ In a Phase II randomized placebo‐controlled trial, the ICP‐lowering capability of exenatide was analyzed. Patients with active IIH underwent surgical implantation of a telemetric ICP monitor and were treated with subcutaneous exenatide or a matched placebo. The administration of exenatide caused a considerable decrease in ICP compared to placebo at 2.5 h (−5.7 ± 2.9 cmCSF; *p* = 0.048), 24 h (−6.4 ± 2.9 cmCSF; *p* = 0.030), and 12 weeks (−5.6 ± 3.0 cmCSF; *p* = 0.058) after chronic use.^158^ In addition, GLP‐1RAs have been shown to improve menstrual regularity and increase fertility rates in females with polycystic ovary syndrome with overweight or obesity; however, this effect has not yet been examined in the IIH population.^159^ Taken together, these novel targeted therapies represent promising approaches for the treatment of IIH; however, additional research is essential to assess their efficacy and impact fully.

### 6.3 Surgical selection

Surgical approaches used for IIH management include CSF diversion, optic nerve sheath puncture, and venous sinus stenting. The guidelines suggest that individuals with sight‐threatening conditions or medically refractory IIH should undergo surgical intervention.^6^

Optic nerve sheath fenestration (ONSF) effectively alleviates intraocular pressure by directly excising the optic nerve sheath to facilitate CSF drainage, thus protecting the optic nerve.^160^ ONSF can relieve papilledema in both eyes and improve visual acuity and visual field impairment caused by IIH, and it can be used when other CSF diversion procedures or medical therapies have failed.^161^, ^162^ Although opinions vary on the best time for surgery, initiating ONSF at an early stage can significantly prevent vision deterioration. CSF shunting reduces compression of the peripheral arteries and veins of the optic nerve by diverting the CSF into the abdominal cavity. It mainly involves two surgical methods: ventriculoperitoneal shunting and lumbar peritoneal shunting. The effectiveness of CSF shunting is well‐established; the overall rates of improvement in headache, papilledema, and visual impairment are 91%, 96%, and 85%, respectively.^163^ However, this intervention is associated with a relatively high revision rate (42% according to a meta‐analysis) and potential complications, including infection, hypotension, headache, and subdural hematoma.^163^, ^164^

In the past decade, cerebral venous sinus stenting (VSS) has become increasingly prevalent. From 2016 to 2020, the number of VSS procedures performed in the United States has increased by 80% annually. The number of CSF shunts decreased by 19%, and that of ONSF procedures decreased by 54%.^165^ Recent findings from single‐center and small multicenter studies have indicated that VSS effectively reduces ICP, preserves vision, and alleviates headache or pulsatile tinnitus in patients with IIH. Furthermore, VSS demonstrated superior safety as compared to CSF shunting and ONSF.^166^, ^167^, ^168^, ^169^ A meta‐analysis of 20 studies from 18 centers showed that the overall improvements in papilledema, headache, and pulsatile tinnitus rates in patients with IIH treated with VSS were 93.7%, 79.6%, and 90.3%, respectively.^170^ Considering the absence of correlation of the severity of stenosis with hemodynamics, CSF open pressure, and clinical presentation, it is crucial to determine the pressure gradient between the proximal and distal ends of the stenosis by intravenous angiography before conducting sinus stent placement surgery. This serves as a crucial reference for assessing patient eligibility for surgical intervention.^94^, ^171^, ^172^ A systematic review revealed a positive correlation between an increased pressure gradient and favorable clinical outcomes. Patients with a pressure gradient greater than 21 mmHg enjoy a high prognosis rate of 94.2%, as compared to only 82.0% for those with a pressure gradient of ≤21 mmHg.^173^ Although no definitive threshold has been established for the pressure gradient, most studies have employed a threshold of >8–10 mmHg.

To date, the most effective surgical procedure has remained undetermined, and no agreement has been reached worldwide on indications for surgery. The choice of surgical technique is mainly dependent on the hospital's medical proficiency and the practical experience of the physicians. Consequently, appropriately designed randomized controlled trials are essential to assess the relative efficacy and safety of various surgical techniques.

## 7 SEXUAL DIMORPHISM IN IIH

According to available epidemiological data, women are more likely to develop IIH compared to men, and this gender‐specific propensity to the disorder does not appear until after puberty, with some studies reporting that the female‐to‐male ratio for prepubertal patients is close to 1.^174^, ^175^ In addition, several studies have reported differences in age at diagnosis of IIH between men and women, with results suggesting that the mean age of female patients is younger than that of male patients.^16^, ^62^, ^176^ A multicenter retrospective study that included 721 patients with IIH explored differences in clinical presentation between men and women and found that men were more likely to have visual impairment (35% vs. 20%, *p* = 0.005) and were at higher risk of severe vision loss.^62^ Similarly, Michael Wall et al. found that the male gender was a potential risk factor for progressive visual field defects.^177^

Some investigations have theorized that the action of sex hormones on CSF dynamics could be responsible for variations in ICP. Israelsen IME et al. hypothesized that alterations in hormonal composition affect the activity of membrane proteins involved in the secretion of CSF at CP.^178^ Based on this hypothesis, they investigated the differences in the expression of various transporters critical for CSF secretion at CP between male rats and female rats under varying estrous cycle conditions.^178^ The findings indicated that estrous female rats exhibited a decrease in the expression of AQP‐1 and carbonic anhydrase isozyme II compared to male rats, while the expression of NKCC1 and carbonic anhydrase isozyme III was higher. In particular, no significant differences were observed between male rats and metestrus female rats, suggesting that transporter expression at CP is sexually differentiated and influenced by the estrous cycle stage.^178^ It is necessary to explore whether the expression of these proteins is also sex‐dependent in IIH and how it affects CSF homeostasis in additional studies.

The use of transorbital sonography to measure optic nerve sheath diameter (ONSD) for noninvasive ICP monitoring is gaining widespread acceptance and popularity. Patrick Goeres's research found a gender difference in mean ONSD among a healthy population, with males having an average of 3.80 and females having an average of 3.54 (*p* = 0.0001).^179^ An observational study by Jakob Pansell et al. aimed to investigate sex‐based differences in the accuracy of ONSD as an indicator of elevated ICP. The results showed that the area under the receiver operator characteristics curves in women ranged from 0.70 to 0.83, while for men, none exceeded 0.5 significantly. This suggests that there is a significant correlation between ONSD and ICP in women, but this is not the case for men.^180^ A hypothesis that has yet to be proven is that the thicker perioptic dura mater and the more efficient drainage of perioptic CSF and glymphatic fluids in men, compared to women, may be the reason why ONSD is less sensitive to changes in ICP in men.^180^ While Jakob Pansell et al. conducted the first study to explore the relationship between gender and ONSD diagnostic accuracy, patients in their study were mainly those with increased ICP resulting from secondary factors. Therefore, despite studies that have now confirmed the diagnostic utility of ONSD in IIH, future evaluations remain needed to determine if the optimal threshold should be adjusted according to gender.^181^

Sex‐based differences in terms of neurovascular functions, pathological brain lipid metabolism, and response to disease‐modifying therapies have been identified in a range of nervous system diseases, including ischemic stroke, Alzheimer's disease, Parkinson's disease, and migraine.^182^, ^183^, ^184^, ^185^ Complex interactions between hereditary elements and sex hormones may account for the different susceptibility to diseases in men and women. There is a lack of research on the impact of sexual dimorphism on IIH; further in‐depth and systematic research is necessary to unravel the underlying pathological processes specific to gender and to more effectively pinpoint potential therapeutic interventions. Clinicians should recognize the critical role of sex differences in IIH and take into account the potential sexual dimorphism in neuroprotective responses to realize the goal of personalized treatment for patients.

## 8 CONCLUSION

Although IIH is considered a rare disease, its incidence is increasing, and correlates with the increasing incidence of obesity worldwide. In addition to headaches and visual impairment, the spectrum of clinical manifestations and comorbidities in individuals with IIH is expanding. These include impaired fertility, complications during pregnancy, OSA, reduced cognitive function, and various adverse mental health outcomes.^63^, ^186^, ^187^, ^188^, ^189^ In 2014, Chen and Wall categorized the numerous risk factors for IIH identified in previous studies into four categories: (1) highly likely risk factors: female sex, obesity/weight gain, endocrine disorders, etc.; (2) probable risk factors: hormone replacement therapy, tetracycline and its derivatives, etc.; (3) possible risk factors: sleep apnea, systemic lupus erythematosus, iron deficiency anemia, etc.; and (4) unlikely or unproven risk factors: oral contraceptive use, pregnancy, arterial hypertension, etc.^190^ Individuals with IIH have an abnormal metabolome, unique androgen signature, and dysregulated glucocorticoid phenotype.^113^, ^191^ Growing evidence suggests that IIH may not be purely idiopathic but is related to systemic metabolic and hormonal disturbances. The early diagnosis and treatment of IIH still pose challenges.

Weight loss is currently the only disease‐modifying therapy, and bariatric surgery provides long‐term ICP reduction.^143^ Adherence to weight loss therapy can be challenging due to its highly restrictive nature. Recent pathophysiological discoveries have led to the development of innovative targeted therapies: 11β‐HSD1 inhibitors and GLP‐1RAs may emerge as potent medications for effectively mitigating intracranial hypertension and improving metabolism. Treatment of IIH as a systemic metabolic disease requires a multidisciplinary strategy for comprehensive management. Understanding the etiology and pathogenesis of IIH is vital to ensuring its early diagnosis and treatment. Current research has made significant progress, and treatment modalities are constantly evolving. However, the long‐term efficacy and safety of these therapeutic approaches remain debated, highlighting the need for further investigation and clinical validation.

## AUTHOR CONTRIBUTIONS

XMJ and CZ: conceptualization (lead), review, and editing (lead). CXZ and YFZ: writing—original draft (lead), formal analysis (lead), review, and editing (equal). LL, HMJ, and HMW: data curation (lead), formal analysis (supporting), writing, reviewing, and editing (supporting). All the authors contributed to the manuscript and approved the submitted version.

## FUNDING INFORMATION

This work is supported by the National Natural Science Foundation of China (82027802), the Pharmaceutical Collaboration Project of the Beijing Science and Technology Commission (Z181100001918026), and the Talents Gathering Project of Xuanwu Hospital Capital Medical University.

## CONFLICT OF INTEREST STATEMENT

The authors declare that the research was conducted in the absence of any commercial or financial relationships that could be construed as a potential conflict of interest.

## Data Availability

The data that support the findings of this study are available from the corresponding author upon reasonable request.
